# Dietary diversity status and influencing factors among children aged 3–7 in China

**DOI:** 10.3389/fnut.2026.1797862

**Published:** 2026-05-25

**Authors:** Bingbing Guo, Lan Jiang, Xinye Jiang

**Affiliations:** Wuxi Maternity and Child Health Hospital, Affiliated Women’s Hospital of Jiangnan University, Wuxi, Jiangsu, China

**Keywords:** children, China, development, dietary diversity scores, influencing factors

## Abstract

**Objective:**

To investigate the dietary diversity status of preschool children aged 3–7 years in Wuxi City, analyze the factors influencing dietary diversity, and provide a scientific basis for developing effective intervention methods to improve dietary diversity.

**Methods:**

A stratified cluster random sampling method was used to select 3,727 preschool children from 18 kindergartens across Wuxi. A questionnaire was used to collect demographic data of the children and their parents, dietary habits, and family conditions.

**Results:**

The average dietary diversity score (DDS) was 5.07 ± 1.94, with 36.79% of children having low dietary diversity (DDS ≤ 4). Multivariate logistic regression analysis indicated that the following factors were associated with normal dietary diversity: family per capita monthly income of 1,000–2,000 RMB, children engaging in outdoor activities for less than 2 h daily or more than 2 h daily, an average daily sleep duration of 9–13 h, occasional good appetite, sometimes good appetite, often good appetite, always good appetite, and having a fixed dining seat. Conversely, factors associated with low dietary diversity included maternal BMI of 24–27.9 (OR = 0.725, 95%*CI*: 0.539 ~ 0.975, *p* = 0.034), maternal BMI > 28 (OR = 0.649, 95%*CI:* 0.483 ~ 0.872, *p* = 0.004), and daily screen time of >1 h (OR = 0.700, 95%*CI:* 0.523 ~ 0.935, *p* = 0.016), 1–2 h (OR = 0.622, 95%*CI:* 0.459 ~ 0.843, *p* = 0.002), or >2 h (OR = 0.620, 95%*CI:* 0.428 ~ 0.900, *p* = 0.012).

**Conclusion:**

Over one-third of preschool children in Wuxi have low dietary diversity, which requires greater attention. Screen time was significantly associated with low dietary diversity, Maternal overweight/obesity was significantly associated with lower dietary diversity among children. Particular focus should be placed on children with prolonged screen time and maternal overweight/obesity to prevent diseases related to low dietary diversity. Targeted intervention strategies for Wuxi’s preschool children were proposed from the perspectives of family, kindergarten and government, and stratified interventions were suggested for migrant children and children with maternal obesity.

## 1 Introduction

Dietary diversity refers to the intake of a variety of different food types, ensuring that the body receives a balanced supply of nutrients, energy, and micronutrients essential for maintaining good health. It is a critical factor for growth and development, particularly for preschool children who need a wide range of nutrients for healthy development. Low dietary diversity, characterized by a lack of food variety and quantity, can result in deficiencies in several nutrients and trace elements (1). In developing countries, the primary nutritional problems affecting children’s health stem from a lack of both the quantity and variety of food, particularly a low intake of vegetables and fruits (2). Children in these regions typically rely on cereals as their primary source of nutrients, often lacking high-quality proteins and fruits (2, 3). Furthermore, research has shown that dietary diversity is influenced by cultural, economic, and transportation factors, as well as local environmental and financial support (4). To better evaluate dietary quality, researchers have employed dietary diversity scores (DDS) (5), which are widely used to assess the nutritional status of various populations and age groups (6, 7).

Low dietary diversity is often associated with malnutrition-related diseases such as stunted growth, dyslipidemia, cardiovascular risks, and metabolic syndrome (8, 9). Children with higher dietary diversity are more likely to have good health outcomes (8). Globally, nearly 200 million children under the age of five suffer from stunted growth, wasting, or both. The World Health Organization (WHO) reported in 2022 that nearly one-third of children in some underdeveloped countries experience stunted growth (10), with the primary causes being food scarcity or a lack of dietary diversity (11). With China’s rapid economic development, dietary patterns have evolved, leading to an increase in nutrient consumption and a reduction in malnutrition-related diseases. However, childhood obesity is emerging as a significant nutritional issue. According to a report released by the Chinese Center for Disease Control and Prevention in 2019, the prevalence of overweight and obesity among school-aged children was 10.88 and 7.87%, respectively (12, 13). Scholars have linked the increased intake of fats and carbohydrates among preschool children to the rise in overweight and obesity (14–16). Excessive consumption of fats and carbohydrates is often associated with low dietary diversity, characterized by the dominance of ultra-processed, high-sugar, and high-fat foods in children’s diets, leading to obesity (17). Without timely and effective intervention, the number of overweight and obese children in China could exceed 40 million by 2030 (18, 19). Thus, low dietary diversity not only impacts issues like stunted growth and malnutrition but also indirectly contributes to the obesity problem (20).

Currently, research on the dietary patterns and food variety of Chinese preschool children is relatively scarce, with most studies focusing on nutritional status assessments or the effects of interventions on children’s nutritional status. However, dietary diversity, as a key indicator of diet quality, is under-researched in this population, especially for preschool children in the Yangtze River Delta region. This study focuses on preschool children in Wuxi, a typical city in the Yangtze River Delta, and for the first time reveals the association chain of “maternal BMI-screen time-dietary diversity” and the special dietary diversity characteristics of local low-income families. It fills the gap in research on dietary diversity influencing factors of urban preschool children in the Yangtze River Delta, and provides a scientific basis for effective nutrition education and targeted interventions.

## 2 Methods

### 2.1 Study population

Wuxi City has a total of approximately 167,300 preschool children aged 3–7 years in 2024, including 137,103 urban registered children and 30,197 migrant children, with a male to female ratio of about 1.08:1(Data sourced from the 2024 Wuxi Statistical Communique on National Economic and Social Development). In August 2024, a stratified cluster random sampling method was used to select guardians of children attending kindergartens in 8 urban districts of Wuxi. A total of 18 urban public and private kindergartens were selected, and valid questionnaires were collected from 3,727 guardians. The age distribution of the study sample was consistent with the overall distribution of preschool children in Wuxi (*χ^2^* = 2.36, *p* = 0.66), and the migrant population accounted for 18.50% of the sample, ensuring the representativeness of the sample in terms of socioeconomic diversity.

#### 2.1.1 Inclusion criteria and non-inclusion criteria

Participants were preschool children aged 3–7 years enrolled in kindergartens in Wuxi City. Eligibility also required that their guardians were capable of independently completing the online questionnaire and provided written informed consent for voluntary participation in the study. Children were non-included if they had congenital disorders, chronic medical conditions, or food allergies; if they had suffered from acute illnesses such as cold or fever within the preceding month; or if their guardians were unable to reliably report information on the children’s dietary intake and lifestyle behaviors.

### 2.2 Survey methods and content

A self-designed questionnaire, based on the multi-indicator cluster survey developed by UNICEF and combined with the dietary characteristics of Chinese preschool children, was employed. This questionnaire was deemed highly reliable and valid (21) The survey was conducted online via QR code distribution. The questionnaire collected information on the child’s gender, age, height, weight, birth weight, and parental information (maternal/paternal weight, height, education level, maternal age during pregnancy; maternal height and weight were self-reported by guardians). Household data, such as monthly per capita income, family size, primary caregiver type and household registration type, were also gathered. The 24-h dietary intake and the child’s usual dietary behaviors and lifestyle habits (outdoor activity time, screen time, sleep duration, appetite, dining habits) were recorded.

### 2.3 Evaluation indicators and definitions

#### 2.3.1 Dietary diversity

Defined based on the Guidelines for Measuring Household and Individual Dietary Diversity issued by the Food and Agriculture Organization (FAO) (22). and adapted according to the Chinese Dietary Guidelines for Preschool Children. The adapted tool has been validated (23) and applied in multiple domestic studies on dietary nutrition of preschool children aged 3–7 years (24, 25). Food intake was categorized into nine groups: cereals, tubers, vegetables, fruits, legumes/nuts, meat, aquatic products, eggs, and dairy products. Each food group consumed in the past 24 h was assigned 1 point, with a maximum score of 9. A score ≤4 was defined as low dietary diversity (22).

#### 2.3.2 BMI Calculation

Body Mass Index (BMI) was calculated as follows:

$$\mathrm{BMI}=\text{Weight}\;(\mathrm{kg})/\text{Height}\;{\left(m\right)}^{2}$$

#### 2.3.3 Pregnancy-related Information

Maternal weight gain during pregnancy was defined as the weight difference before and after pregnancy. Gestational complications included conditions like hypertension, diabetes, hyperlipidemia, viral hepatitis, and thyroid dysfunction. Low birth weight was defined as <2.5 kg, and macrosomia as ≥4 kg.

### 2.4 Quality control

Before the study commenced, a feasibility analysis was conducted involving kindergarten teachers, maternal and child healthcare staff, and guardians. This ensured that the survey could be carried out smoothly. The questionnaire was designed with selectable options for each question to avoid missing data, and logical error checks were included to flag any input discrepancies. During data processing, two independent investigators entered and reviewed the data, with discrepancies resolved by a third party.

### 2.5 Statistical analysis

Statistical analyses were performed using SPSS 19.0. Continuous variables were presented as mean ± standard deviation (x̅±s), while categorical variables were expressed as percentages (*n*%). Chi-square tests were used for comparisons between groups. Variables with *p* < 0.05 in the univariate analysis were included in the initial model of multivariate logistic regression analysis, and the forward stepwise regression method was used to screen variables, with *p* < 0.05 as the inclusion criterion for the final model. Before regression analysis, the variance inflation factor (VIF) was used to test the multicollinearity of independent variables. Categorical variables were coded as follows: unordered categorical variables were coded with dummy variables, and ordered categorical variables were coded with rank variables, with the lowest/reference group as 0. Statistical significance was set at *p* < 0.05. Variable coding is shown in Table 1.

**Table 1:** Variable coding.

Variable	Coding
Low dietary diversity (DDS ≤ 4)	0 = No, 1 = Yes
Maternal BMI	0 = <18.5, 1 = 18.5–23.9, 2 = 24–27.9, 3 = > 28
Maternal age at pregnancy	0 = ≤35, 1 = > 35
Number of children in the family	0 = 1 child, 1 = 2 children, 2 = 3 children
Maternal education level	0 = Elementary or lower, 1 = Middle school, 2 = High school or vocational, 3 = College, 4 = Bachelor’s, 5 = Master’s or higher
Paternal education level	Same as maternal education level
Monthly household income (CNY)	0 = <1,000, 1 = 1,000–2000, 2 = 2000–5,000, 3 = 5,000–10,000, 4 = > 10,000
Outdoor activity time (per day)	0 = Rarely, 1 = Regularly but <2 h, 2 = > 2 h
Screen time (per day)	0 = <15 min, 1 = 15–30 min, 2 = 30–60 min, 3 = > 1 h, 4 = 1–2 h, 5 = > 2 h
Sleep duration (per day)	0 = <9 h, 1 = 9–13 h, 2 = ≥13 h
Child’s appetite	0 = Poor, 1 = Occasionally good, 2 = Sometimes good, 3 = Often good, 4 = Always good
Fixed dining seat	0 = No, 1 = Yes
Independent eating utensils	0 = No, 1 = Occasionally, 2 = Sometimes, 3 = Often, 4 = Always

## 3 Results

### 3.1 General characteristics

A total of 3,727 valid questionnaires were collected, covering preschool children from 18 kindergartens across Wuxi City. The children’s ages ranged from 3 to 7 years, with an average age of 5.35 ± 0.98 years. The sample comprised 1924 boys (51.62%) and 1803 girls (48.38%). The mean dietary diversity score (DDS) was 5.07 ± 1.94, and the most frequently consumed food categories were starchy staples (96.54%), dairy products (78.19%), and fruits (73.09%). The least consumed food groups were tubers (19.32%), aquatic products (21.01%), and legumes/nuts (21.79%) (see Table 2).

**Table 2:** Dietary diversity of preschool children.

Food category	Male (*n* = 1924)	Female (*n* = 1803)	Total (*n* = 3,727)
Starchy staples	1859 (96.62%)	1739 (96.45%)	3,598 (96.54%)
Tubers	383 (19.91%)	337 (18.69%)	720 (19.32%)
Vegetables	1,359 (70.63%)	1,284 (71.21%)	2,643 (70.91%)
Fruits	1,387 (72.09%)	1,337 (74.15%)	2,724 (73.09%)
Meat or organ meat	1,290 (67.05%)	1,168 (64.78%)	2,458 (65.95%)
Aquatic products	424 (22.04%)	359 (19.91%)	783 (21.01%)
Eggs	1,175 (61.07%)	1,070 (59.35%)	2,245 (60.24%)
Legumes, nuts, seeds	455 (23.65%)	357 (19.80%)	812 (21.79%)
Dairy products	1,506 (78.27%)	1,408 (78.09%)	2,914 (78.19%)

### 3.2 Univariate analysis of low dietary diversity

The prevalence of low dietary diversity (DDS ≤ 4) among preschool children in Wuxi was 36.79% (1,371/3727). Significant differences were observed in the prevalence of low dietary diversity across several factors, including maternal BMI, maternal age at pregnancy, number of children in the family, parental education level, household income, outdoor activity time, screen time, sleep duration, use of independent eating utensils, fixed dining seat, and child’s appetite (*p* < 0.05). Children with obese or overweight mothers, older mothers, less educated parents, lower household income, less outdoor activity, and longer screen time were more likely to have low dietary diversity. Variables with *p* > 0.05 (gender, age, birth weight, Paternal BMI, delivery method, pregnancy complications, caregiver type) were excluded from subsequent multivariate analysis (see Tables 3, 4).

**Table 3:** Univariate analysis of low dietary diversity by socio-demographic characteristics.

Factors	Total number	Number with low dietary diversity	Low dietary diversity rate (%)	χ^2^	*p*
Maternal BMI				18.154	<0.001
<18.5	348	100	28.74		
18.5–23.9	2,152	782	36.34		
24–27.9	602	225	37.38		
>28	624	264	42.31		
Maternal age during pregnancy				8.969	<0.001
≤35	3,475	1,256	36.14		
>35	250	114	45.60		
Number of children				17.463	<0.001
1	2,299	791	34.41		
2	1,377	565	41.03		
3	51	15	29.41		
Maternal education level				53.098	<0.001
Primary school or below	19	12	63.16		
Middle school	340	148	43.53		
High school/vocational school	615	279	45.37		
College	1,067	401	37.58		
Undergraduate	1,541	490	31.80		
Master or above	145	41	28.28		
Paternal education level				37.366	<0.001
Primary school or below	16	10	62.50		
Middle school	299	133	44.48		
High school/vocational school	658	279	42.40		
College	1,119	421	37.62		
Undergraduate	1,475	484	32.81		
Master or above	160	44	27.50		
Monthly household income (RMB)				34.484	<0.001
<1,000	50	25	50.00		
1,000–2000	51	16	31.37		
2000–5,000	546	240	43.96		
5,000–10,000	1,578	611	38.72		
>10,000	1,502	479	31.89		

**Table 4:** Univariate analysis of low dietary diversity by lifestyle and dietary behaviors.

Factors	Total number	Number with low dietary diversity	Low dietary diversity rate (%)	χ^2^	*p*
Outdoor activity time (daily)				40.985	<0.001
Rarely	236	127	53.81		
Often, but <2 h	2,420	903	37.31		
>2 h	1,071	341	31.84		
Screen time (daily)				45.372	<0.001
<15 min	538	178	33.09		
15–30 min	1,291	433	33.54		
30–60 min	1,102	388	35.21		
>1 h	344	155	45.06		
1–2 h	291	136	46.74		
>2 h	161	81	50.31		
Average sleep time (daily, hours)				17.351	<0.001
<9	391	180	46.04		
9–13	3,244	1,153	35.54		
≥13	92	38	41.30		
Children using own tableware				66.508	<0.001
No	179	90	50.28		
Occasionally	196	78	39.80		
Sometimes	334	148	44.31		
Often	654	297	45.41		
Always	2,364	758	32.06		
Fixed dining seat				45.101	<0.001
No	568	280	49.30		
Yes	3,159	1,091	34.54		
Appetite consistency				54.375	<0.001
Poor	298	151	50.67		
Occasionally	599	240	40.07		
Sometimes	1,199	468	39.03		
Often	1,122	370	32.98		
Always	509	142	27.90		

### 3.3 Multivariate analysis of factors influencing low dietary diversity

Variables that were statistically significant in the univariate analysis were included in the multivariate logistic regression analysis model. Multicollinearity test showed that all VIF values of variables were <10, with no serious multicollinearity. Potential interaction terms (e.g., income × maternal BMI, screen time × outdoor activity time) were included in the model for testing, and all interaction terms had *p* > 0.05 with no statistical significance, so they were not included in the final model. The results showed that factors such as a monthly household income of 1,000–2000 CNY, regular but less than 2 h of daily outdoor activity, an average daily sleep duration of 9–13 h, a good appetite, and having a fixed dining seat were associated with an increased likelihood of normal dietary diversity. In contrast, maternal overweight or obesity and longer daily screen time were associated with a higher risk of low dietary diversity. Variables with *p* > 0.05 (maternal age at pregnancy, number of children, maternal/paternal education level, children using own tableware) were excluded from the final model (see Table 5).

**Table 5:** Multivariate logistic regression analysis of factors influencing low dietary diversity.

Factors	B	Wals	*P*	OR	95% C.I.	
					Lower	Upper
Maternal BMI						
<18.5				1.000		
18.5–23.9	−0.305	5.398	0.054	0.803	0.569	1.024
24–27.9	−0.322	4.512	0.034	0.725	0.539	0.975
>28	−0.433	8.220	0.004	0.649	0.483	0.872
Monthly household income (RMB)						
<1,000				1.000		
1,000–2000	1.012	5.183	0.023	2.752	1.151	6.579
2000–5,000	0.263	0.697	0.404	1.301	0.701	2.413
5,000–10,000	0.372	1.473	0.225	1.450	0.796	2.643
>10,000	0.594	3.733	0.053	1.811	0.991	3.307
Outdoor activity time						
Rarely				1.000		
Often, but <2 h	0.432	8.952	0.003	1.540	1.161	2.043
>2 h	0.673	19.170	0.000	1.961	1.451	2.651
Screen time (daily)						
<15 min				1.000		
15–30 min	0.036	0.100	0.751	1.036	0.831	1.293
30–60 min	−0.036	0.098	0.755	0.965	0.769	1.210
>1 h	−0.357	5.812	0.016	0.700	0.523	0.935
1–2 h	−0.475	9.354	0.002	0.622	0.459	0.843
>2 h	−0.477	6.344	0.012	0.620	0.428	0.900
Average sleep time (daily, hours)						
<9				1.000		
9–13	0.235	4.219	0.040	1.265	1.011	1.584
≥13	−0.148	0.357	0.550	0.863	0.531	1.400
Appetite consistency						
Poor				1.000		
Occasionally	0.357	5.727	0.017	1.429	1.067	1.914
Sometimes	0.370	7.407	0.006	1.447	1.109	1.889
Often	0.510	13.498	0.000	1.666	1.269	2.187
Always	0.726	20.554	0.000	2.066	1.510	2.827
Fixed dining seat						
No				1.000		
Yes	0.338	11.160	0.001	1.402	1.150	1.709

## 4 Discussion

This study revealed that the dietary diversity of preschool children in Wuxi is relatively low, with a significant portion of their diet consisting mainly of starchy foods, while the intake of aquatic products and legumes is minimal. This finding may be related to China’s agricultural background, where cereals remain a major source of energy and nutrients. However, cereals such as rice and flour are deficient in lysine, an essential amino acid, contributing to the overall lower nutritional quality of the diet.

As China’s economy continues to develop and foreign cultural influences increase, dietary patterns have evolved, especially with the popularity of foods like hamburgers and pasta. This has affected the composition of children’s food intake, leading to a reduction in the consumption of high-quality foods like aquatic products, legumes, vegetables, and fruits, which results in widespread micronutrient deficiencies, particularly in vitamins A, B, C, and minerals (26). Studies have also found a positive correlation between nutritional patterns and children’s growth and development (27). Therefore, according to the *Chinese* Dietary Nutrient Reference Intake standards, preschool children’s diets should include more legumes, aquatic products, and tubers to improve dietary diversity, reduce the risk of nutrient deficiency-related chronic diseases (28, 29), and address issues of overweight and obesity.

The dietary diversity score (DDS) quantifies the variety of food groups consumed and serves as an effective measure of micronutrient intake (30). A higher DDS indicates a richer intake of nutrients, which positively affects children’s physical development (24). Several international studies have shown that children with higher DDS have better growth outcomes (8, 31). In this study, the average DDS of 3-7-year-old children was 5.07, lower than that reported in other studies of Chinese children. For instance, Bi et al. (32) reported a DDS of 5.77, Meng et al. (24) reported 6.1, Zhao et al. (30) reported 6.8, and Jiang et al. (25) reported 7.4. However, it was higher than the DDS of 4.18 reported for Chinese children aged 2–17 years (33). These discrepancies may be attributed to regional, ethnic, and economic differences, as well as dietary habits in rural areas and among minority groups, which tend to lower dietary diversity. Additionally, variations in measurement methods or recall bias may influence DDS results. Some studies have found that increasing DDS by 1% can reduce the risk of poor health outcomes in children by 10%. Inadequate dietary diversity not only adversely affects children’s health but also imposes a significant economic burden on governments and families in developing countries (34). Thus, by analyzing the factors influencing dietary diversity, including children’s eating habits, family environment, and parental factors, this study aims to identify key influencers of dietary diversity and recommend appropriate policies to promote diverse diets in children.

The study found that family per capita monthly income of 1,000–2000 RMB was associated with higher dietary diversity, which was different from the common conclusion that higher income is associated with higher DDS (35, 36). This is because the sample size of this income group is small (*n* = 51), mainly consisting of local low-income families in Wuxi, who enjoy free nutrition guidance from maternal and child health institutions and preschool children’s dietary subsidies provided by the local government. These families have a high attention to children’s dietary collocation and follow the nutritional guidance to ensure the variety of children’s food. In contrast, the 2000–5,000 RMB income group is mostly migrant families, whose irregular living and eating habits lead to low dietary diversity of children; the >10,000 RMB high-income group has the problem of excessive intake of snacks and ultra-processed foods for children, which reduces the dietary diversity of children. This result suggests that economic income is not the only factor affecting dietary diversity, and targeted nutritional intervention can effectively improve dietary diversity even for low-income families. Adequate sleep duration and regular outdoor activity also increased the likelihood of higher dietary diversity in children, which is consistent with a Chinese community-based study that found a positive correlation between adequate sleep and DDS (37). During the preschool years, while growth slows compared to infancy, overall development remains rapid, and the need for a variety of nutrients increases. Adequate sleep supports proper physiological function, enhancing children’s ability to consume a diverse range of foods, in contrast to insufficient or excessive sleep. Additionally, regular outdoor activity stimulates children’s appetites, encourages timely meals, and reduces the likelihood of irregular eating patterns. Furthermore, having a fixed dining seat was found to increase dietary diversity, as it fosters consistent mealtime habits and reduces the intake of ultra-processed foods, high-sugar snacks, or late-night meals, which are often associated with lower dietary diversity.

On the other hand, maternal overweight or obesity was associated with lower dietary diversity in children. Maternal height and weight were self-reported in this study, which may have certain bias, but the results are still reliable. Maternal BMI is an important proxy variable for family unhealthy dietary patterns. Maternal overweight or obesity, unless caused by disease, is often influenced by unhealthy eating habits, such as low dietary diversity. Obesity and unhealthy dietary patterns tend to cluster in families, and long-term exposure to low dietary diversity increases the risk of obesity in children under maternal care. A longitudinal study in the United States found a negative correlation between weight gain and the intake of fruits and vegetables (38), further reducing dietary diversity. Excessive screen time was also linked to lower dietary diversity, as it reduces physical activity and promotes snacking and the consumption of high-sugar, high-fat convenience foods, which detract from a balanced diet.

### 4.1 Study limitations

This study has several limitations that need to be explicitly acknowledged: The survey data were all from self-reported questionnaires by guardians, which may have recall bias and social desirability bias, such as underreporting of screen time and overreporting of outdoor activity time. The dietary assessment tool was adapted based on FAO guidelines and domestic dietary guidelines, and has been validated in other domestic studies, but not independently validated for the study population in Wuxi. There are potential residual confounding factors in the study, such as parents’ nutritional knowledge level, children’s dietary habit formation time, and family cooking methods, which were not included in the regression model. Screen time and outdoor activity time were self-reported by guardians, which may have misclassification bias. Appetite may be endogenous to dietary diversity, and the two may influence each other, and the cross-sectional design cannot determine the direction of the association. The study only assessed the diversity of food categories, and did not evaluate the food portion size and nutrient adequacy, which cannot fully reflect the children’s nutritional status. The income groups were divided from continuous variables, and the sample size of each group was unbalanced (e.g., the 1,000–2000 RMB group had only 51 samples), which may affect the interpretation of the results. The study was limited to urban preschool children in Wuxi, and did not include rural children, so the results cannot be generalized to all preschool children in Wuxi or the whole country.

### 4.2 Future research suggestions

Future studies should expand the research scope to include rural preschool children in Wuxi, and conduct a multicenter cohort study covering multiple cities in the Yangtze River Delta to improve the generalizability of the results; use a longitudinal design to explore the causal relationship between influencing factors and dietary diversity, and clarify the causal direction between appetite and dietary diversity; combine objective measurement methods (e.g., accelerometer to measure screen time/outdoor activity time, 24-h dietary recall combined with food weighing to assess dietary intake) to reduce the bias of self-reported data; include more potential confounding factors (e.g., parents’ nutritional knowledge, family cooking methods) in the model to improve the accuracy of the analysis; and evaluate the dietary diversity combined with food portion size and nutrient adequacy to comprehensively reflect the nutritional status of preschool children.

## 5 Conclusion

Greater attention should be paid to dietary diversity among preschool children in Wuxi, as over one-third fail to meet adequate dietary diversity levels. Maternal overweight/obesity was significantly associated with lower dietary diversity among children, Screen time was significantly associated with low dietary diversity, and reducing screen time can be an actionable intervention target. Particular focus should be placed on children with prolonged screen time and a family history of maternal obesity to prevent diseases associated with low dietary diversity. Based on the study results and the characteristics of preschool children in Wuxi, targeted and stratified intervention strategies are proposed from the perspectives of family, kindergarten and government:

### 5.1 Family level

First, control children’s daily screen time to less than 1 h, and increase outdoor activity time to more than 2 h daily to stimulate children’s appetite and improve dietary diversity; In addition, cultivate children’s good dining habits, including fixed dining seats and long-term use of independent eating utensils, to form regular eating patterns; Furthermore, for families with maternal overweight/obesity, synchronously improve the overall family dietary pattern, reduce the intake of high-fat and high-sugar foods, and increase the consumption of vegetables, fruits, aquatic products and legumes; Finally, migrant families should pay attention to the regularity of children’s diet, and avoid the impact of irregular living on children’s dietary collocation.

### 5.2 Kindergarten level

First, integrate dietary diversity education into preschool education courses, carry out “nutrition small classroom” and food cognition activities to improve children’s acceptance of various foods; Second, provide meals in accordance with the Chinese Dietary Guidelines for Preschool Children, ensure that the daily food types cover more than 7 categories, and increase the proportion of aquatic products, legumes and tubers; Furthermore, conduct one-on-one nutritional guidance for the families of migrant children and children with maternal obesity, and provide personalized dietary collocation suggestions; Ultimately, organize outdoor sports activities regularly to increase children’s outdoor activity time in the kindergarten.

### 5.3 Government level

First, continue to provide nutritional meal subsidies and free nutritional guidance for low-income preschool children families in Wuxi, and expand the coverage of nutritional intervention policies; Second carry out parental nutritional knowledge training, focusing on families with maternal overweight/obesity and migrant families, and popularize the importance of dietary diversity and scientific dietary collocation methods; Moreover, formulate the assessment standards for dietary diversity of kindergarten meals in Wuxi, and conduct regular supervision and evaluation to ensure the nutritional quality of kindergarten meals; Finally, establish a long-term monitoring system for the dietary diversity of preschool children in Wuxi, and dynamically grasp the changes of dietary diversity status and influencing factors.

## Data Availability

The raw data supporting the conclusions of this article will be made available by the authors, without undue reservation.
